# Rapid deprotection and purification of ceftaroline free base from its phosphoramidate prodrug

**DOI:** 10.1099/jmm.0.002077

**Published:** 2025-09-17

**Authors:** Souvik Roy, Robert D. Kina, Nathaniel N. Koloian, Daniah Zubair, Alexis A. Brecht, Andrew M. Lipchik, Andrew D. Berti

**Affiliations:** 1 Department of Pharmaceutical Sciences, Eugene Applebaum College of Pharmacy and Health Sciences, Wayne State University, Detroit, MI 48201, USA; 2 Department of Pharmacy Practice, Eugene Applebaum College of Pharmacy and Health Sciences, Wayne State University, Detroit, MI 48201, USA; 3 Department of Biochemistry, Microbiology and Immunology, School of Medicine, Wayne State University, Detroit, MI 48201, USA

**Keywords:** ceftaroline, deprotection, phosphoramidate, prodrug

## Abstract

Contemporary pharmaceutical design often incorporates functional groups to improve the pharmacokinetic/pharmacodynamic profile of a desired active chemical entity. These compounds are known as prodrugs. While prodrug enhancements may improve a medication’s clinical utility, they often limit the ability for *in vitro* testing of the active drug. Published protocols suggest that commercially available phosphatase enzymes can provide a straightforward and cost-effective way to access the active components of phosphoramidate prodrugs. Here, we demonstrate that commercial phosphatases lack reproducibility in generating the active antibiotic ceftaroline from its prodrug ceftaroline fosamil (Teflaro^®^). We propose that previously reported successes with phosphatase-mediated conversion are due to the purification of natural ceftaroline fosamil degradation products or batch-dependent isozyme contaminants present in alkaline phosphatases obtained from biological sources. Here, we demonstrate the chemical/thermodegradation to provide a robust, non-enzymatic source of the ceftaroline free base. This efficient method can be readily adapted to expand the availability of deprotected thermostable commercial pharmaceutical compounds for *in vitro* testing and research purposes.

**Impact Statement**

Anti-infective research and antimicrobial stewardship have made significant advances in addressing new and emerging diseases. Recent technological innovations have led to the development of prodrug medications, such as the fifth-generation cephalosporin, Teflaro^®^ (ceftaroline fosamil). However, due to their inactive nature, prodrugs are ineffective during *in vitro* testing. Previous studies have investigated the use of commercially available phosphatase enzymes to convert ceftaroline fosamil into ceftaroline free base, but this approach has proven unreliable. Our strategy employs a forced-degradation, non-enzymatic pathway to generate ceftaroline free base, providing a method that is both efficient and consistently reliable. Consequently, new anti-infective research can adopt this effective protocol, using commercially available Teflaro^®^ (ceftaroline fosamil) to produce ceftaroline free base for *in vitro* testing. This advancement will facilitate better antibiotic selection and enhance efforts in antimicrobial stewardship.

## Data Summary

The authors confirm that all supporting data, code and protocols have been provided within the article or through supplementary data files.

## Introduction

Prodrugs are pharmacologically inactive compounds that are converted into an active form within the body. They have the potential to provide multiple benefits, including improved bioavailability, enhanced stability, longer half-lives and fewer side effects [1]. The ‘ProTide’ technology has revolutionized the design and delivery of new nucleotide/nucleoside analogue-based antivirals and chemotherapeutics by improving the solubility and bioavailability of these compounds [2]. There is increasing interest in its application to anti-infective agents, specifically to the development of cephalosporins and novel beta-lactamase inhibitors [3,4]. One of the most common prodrug modifications is the phosphoramidation of hydroxy or amino groups [5,6]. Typically, these phosphoramidates are cleaved by either serum or cellular phosphatases to release the active pharmaceutical agent. However, there is wide variation in the activity of individual phosphatases on different phosphoramidate substrates [7].

This past decade has provided numerous examples of FDA approval of new antibiotics without simultaneous approval of an associated commercial susceptibility test to inform appropriate use [8,9]. This was true of the first beta-lactam prodrug in clinical use, ceftaroline fosamil (Teflaro^®^), which was approved for clinical use in October 2010, with susceptibility testing products only becoming commercially available in February 2013 [10,13]. Conventional alternatives, such as broth microdilution testing, are not practical when the antibiotic is a prodrug, necessitating innovative and incomplete solutions such as testing surrogate agents and expanding the capacity of reference laboratory testing [14]. The delay in accurate susceptibility information has profound implications for responsible antimicrobial stewardship and prevention of antibiotic resistance [15]. Furthermore, the lack of active pharmaceutical ingredients limits academic research, which often lays the groundwork for improvements in dosing and expanded indications [16].

Our group had previously used a commercial alkaline phosphatase to successfully generate the ceftaroline free base with excellent yield from Teflaro^®^ clinical powder. However, subsequent attempts by our group and others to repeat the procedure have been inconsistent, potentially due to inter-batch variability in co-purified isozymes, and inspired the current study. Here, we explore several methods to remove the phosphoramidate group from Teflaro^®^ *in vitro* using commercial enzymes or chemical/thermodegradation reactions as a means to further purify the active pharmaceutical ingredient. This proof-of-principle protocol may extend to additional phosphonamino or phosphonamidate-like groups and can be adapted to generate the active form of future phosphoramidate-protected prodrugs, particularly those for which robust forced degradation product characterization is available.

## Methods

### Theory and implementation

**Fig. 1.**
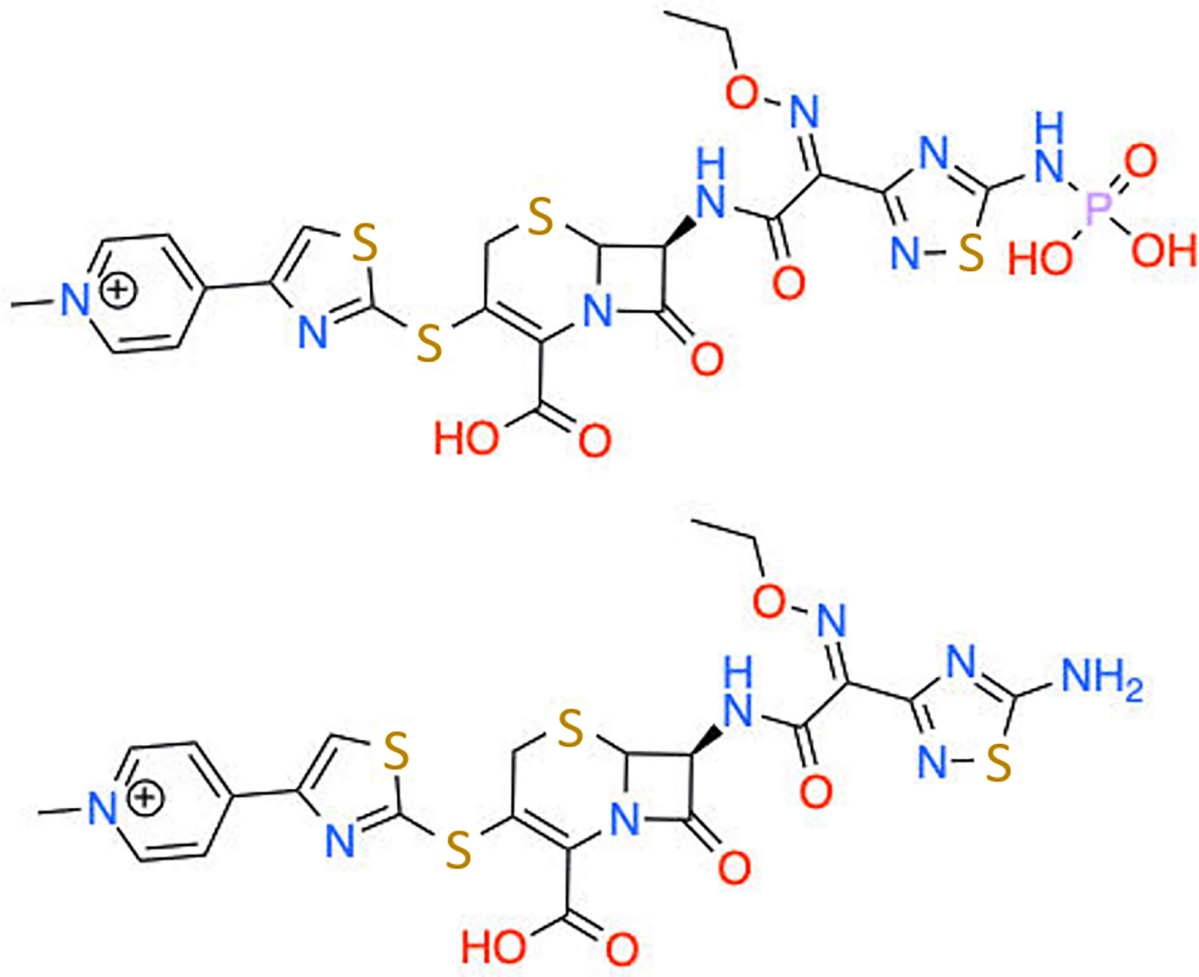
Ceftaroline fosamil (top) is commercially available but biologically inactive. Removal of the phosphoramidate is required to generate the biologically active ceftaroline free base (bottom) to be useful for testing or research purposes.

The ceftaroline free base is insoluble in arginine-buffered aqueous solution; ceftaroline fosamil relies on the *N-*phosphono group of the prodrug for water solubility (Fig. 1) [17]. Therefore, enzymatic removal should yield insoluble ceftaroline free base with all excipients remaining in solution. Chemical/thermodegradation studies of ceftaroline fosamil suggest that the natural degradation product at neutral pH is bona fide ceftaroline free base, and this process can be accelerated with heat. Furthermore, potential side reactions resulting in decomposition or oxidative destruction of the beta-lactam ring would increase aqueous solubility, resulting in nearly pure ceftaroline free base precipitate, which, removed from buffering agents, can be stored as dry powder or resuspended in an appropriate solvent.

### Study materials

Teflaro^®^ was obtained from AbbVie (Wyandotte, MI, NDC 0456-0400). Antarctic phosphatase and Quick CIP were obtained from New England Biolabs (Ipswich, MA, M0289S, M0525S). Bovine intestinal alkaline phosphatase was obtained from Sigma-Aldrich (St. Louis, MO, A2356-10KU). All agents were stored as indicated by the manufacturer before use. Phosphatase activity was confirmed by a minimum 3-log reduction in recovery of nuclease-linearized plasmid DNA treated with phosphatase and subsequently re-ligated using T4 DNA ligase (New England Biolabs, Ipswich, MA, M0202T) per manufacturer’s instructions compared to non-phosphatase-treated controls (data not shown).

### Enzymatic removal of phosphoro and phosphono groups

A total of 32.5 mg of Teflaro^®^, equivalent to 20 mg ceftaroline free base and 9.8 mg excipients, was transferred to low-bind microcentrifuge tubes (Avant Lo-Bind, MidSci, St. Louis, MO) for each condition tested. Bovine intestine phosphatase: Teflaro^®^ was resuspended in 187.5 µl of BIAP buffer (5 mM MgCl_2_, 0.2 mM ZnCl_2_, 10 mM Tris HCl, pH 8.0) plus 12.5 µl of bovine intestinal alkaline phosphatase enzyme. Antarctic phosphatase: Teflaro^®^ was resuspended in 178 µl sterile water for injection supplemented with 20 µl 10× Antarctic phosphatase buffer plus 2 µl Antarctic phosphatase enzyme. Quick calf intestine phosphatase: Teflaro^®^ was resuspended in 178 µl sterile water for injection supplemented with 20 µl 10× CutSmart buffer plus 2 µl Quick CIP enzyme. All reactions were incubated at 37 °C for 1 h. No improvements in recovery were observed by exchanging ZnSO_4_ for ZnCl_2_ or by supplementing with additional zinc in reaction buffers based on the recovery of insoluble precipitates. Slight increases in conversion were observed when incubation time was extended to 24 h.

### Thermal phospho-hydrolysis of ceftaroline fosamil

A total of 32.5 mg of Teflaro^®^, equivalent to 20 mg ceftaroline free base and 9.8 mg excipients, was transferred to low-bind microcentrifuge tubes and resuspended in 200 µl sterile water for injection. Parallel experiments of tubes were incubated at three different temperature values (45 °C, 60 °C and 75 °C) and two different durations of exposure (60 min and 180 min). Dried product recovery was determined by weighing water-insoluble products on an analytical balance (AB104-S, Mettler Toledo, Columbus, OH) following centrifugation (12,000 ***g***, 5 min) and complete solvent removal by lyophilization. Per cent recovery was only calculated if at least 90% of a water-insoluble sample’s peak area was recovered at a retention time consistent with either ceftaroline free base or Teflaro^®^ as determined by LC-MS. Per cent recovery was defined as the ratio of recovered to initial product mass (i.e. 20 mg) multiplied by the integrated peak ratio of ceftaroline free base to the total peak area of ceftaroline free base plus Teflaro^®^.

### Purification of deprotected agents

Aqueous enzymatic or thermal degradation reactions were frozen overnight and then thawed on ice to minimize ceftaroline free base solubility. Ceftaroline free base was collected by centrifugation (12,000 ***g***, 4 °C, 10 min), and the supernatant was carefully removed by pipettor. Powder was either lyophilized or resuspended in 30% DMSO in water and stored frozen until analysis. A molar extinction coefficient was calculated from both the lyophilized product and authentic ceftaroline standard (obtained from prior successful enzymatic deprotection reactions), reconstituted per Clinical and Laboratory Standards Institute (CLSI) recommendations in 30% DMSO in water. Both reconstituted powders exhibited an ε_355_=6661 M^−1^ cm^−1^ (95% CI: 5,511–7811) equivalent to 11 ml mg^−1^ cm^−1^ (95% CI: 9.1–12.9), which was used in subsequent experiments to determine ceftaroline free base concentration in solution.

### Analytical validation of prodrug conversion

Ceftaroline standards or isolated products from thermal hydrolysis were dissolved in DMSO and diluted 1 : 100 in buffer A (0.1% formic acid in water) for LC-MS analysis on a Shimadzu LC-MS 2020. The LC-MS is composed of a Prominence series HPLC coupled to an MS-2020 single-quadrupole mass spectrometer, allowing for the simultaneous determination of sample purity and compound identity. LC analysis was performed at a flow rate of 0.4 ml min^−1^ and a gradient of 5–95% buffer B (100% acetonitrile/0.1% formic acid) in buffer A (0.1% formic acid in water) over 19 min using an Agilent Eclipse Plus C18 column (3.5 µm i.d.×4.6 mm×100 mm). The wavelength of the UV-Vis detector was set to 254 nm. ESI-MS was carried out in positive ion mode with a mass range of m/z 300.0–2000.0, a scan speed of 15,000 u s^−1^, an event time of 0.15 s and a voltage of 4.5 kV. The LC peak areas of ceftaroline fosamil and ceftaroline were transformed into per cent (%) for interpretation and calculation of per cent composition.

### Biological validation of prodrug conversion

Purified ceftaroline powder and extemporaneously prepared Teflaro^®^ prodrug were reconstituted in sterile water for injection and used for broth microdilution MIC testing as outlined by the CLSI [18]. *Staphylococcus aureus* strains ATCC^®^ 29213 and ATCC^®^ BAA-2686 with ceftaroline MICs of 0.5 µg ml^−1^ and 8 µg ml^−1^, respectively [18,19], were obtained from the American Type Culture Collection (Manassas, VA) and used as controls. Plates were supplemented with 10 µg ml^−1^ resazurin 1 h before imaging to enhance visual contrast. Susceptibility readings were recorded prior to the addition of resazurin. Media were supplemented with 100 µg ml^−1^ thiamine to support the growth of ATCC^®^ BAA-2686.

## Results and discussion

### Enzymatic conversion of ceftaroline fosamil to ceftaroline free base

Our initial efforts at reproducing a published bovine intestine phosphatase-based method converting Teflaro^®^ to ceftaroline [20] had limited success with a yield of less than 0.1 mg of insoluble product per 32.5 mg of Teflaro^®^ (<0.5% yield). This was surprising as we had previously used the referenced procedure with excellent results. The phosphatase activity was confirmed, ruling out deactivation of the preparation in transit. The low-yield product was verified to be an active ceftaroline free base by both HPLC-MS analysis and bioassay. While adequate for small-scale susceptibility testing, the high cost of the enzyme and poor yield leave space for assay optimization. Arginine included as excipients in Teflaro^®^ can chelate zinc, potentially limiting the activity of zinc metallophosphatases and explaining the poor recovery. However, supplementation with additional zinc up to 1.5 mM in reaction buffers or altering the metal salt formulation from zinc chloride to zinc sulphate did not improve recovery (less than 0.1 mg of insoluble product per 32.5 mg Teflaro^®^). The specific activity of individual phosphatase enzymes for different phosphoramidate substrates can vary significantly. However, substituting alternative phosphatase enzymes, including one that does not require zinc for catalysis, did not improve the recovery (<0.5% yield for both NEB Antarctic Phosphatase and NEB Quick CIP Phosphatase). Prolonged incubation for 24 h increased recovery to at most 0.5 mg (<2.5% yield), suggesting either a slow dephosphorylation reaction or enzyme-independent degradation to active ceftaroline was occurring.

### Non-enzymatic conversion of ceftaroline fosamil prodrug

After performing a more extensive literature review, we identified a stability-indicating coupled LC-MS assay that reported ceftaroline free base as the major thermal decomposition product of Teflaro^®^ [21]. Reproducing and expanding on the authors’ thermal degradation conditions resulted in significantly improved yields of ceftaroline free base (22±3.7%) without statistically significant differences in recovery at higher temperatures or with longer incubation times (Table 1). Of note, however, prolonged incubation at the highest temperatures (>60 °C) resulted in a bright-red, DMSO-soluble degradation product with an LC-MS profile containing multiple peaks and no detectable ions consistent with pure ceftaroline free base (Fig. S1, available in the online Supplementary Material).

**Table 1.** Forced thermal degradation of Teflaro^®^. Clinical powder equivalent to 20 mg of ceftaroline free base was resuspended in sterile water for injection and incubated at the indicated temperature for the indicated duration. Samples that yielded pale yellow powders were included in a two-tailed ANOVA analysis that failed to demonstrate any significant between-group differences in percentage recovery (*P*=0.2)

Duration (h)	Temp. (°C)	Dried product (mg)	Per cent recovery	Description
1	45	4.7, 3.6, 3.5	20±3.3%	Pale yellow powder
1	60	3.5, 4.8, 4.8	22±3.8%	Pale yellow powder
1	75	7.9, 4.5, 4.6	na	Deep red powder
3	45	4.8, 4.3, 4.0	22±2.0%	Pale yellow powder
3	60	5.7, 5.3, 8.3	28±1.6%	Pale yellow powder*
3	75	8.2, 10.3, 12.4	na	Deep red powder

*The third replicate, incubated at 60 °C for 3 h, exhibited a deep red powder and was not included in percentage recovery or ANOVA calculations.

### High-temperature, short-duration conversion of ceftaroline fosamil prodrug to ceftaroline free base

**Fig. 2.**
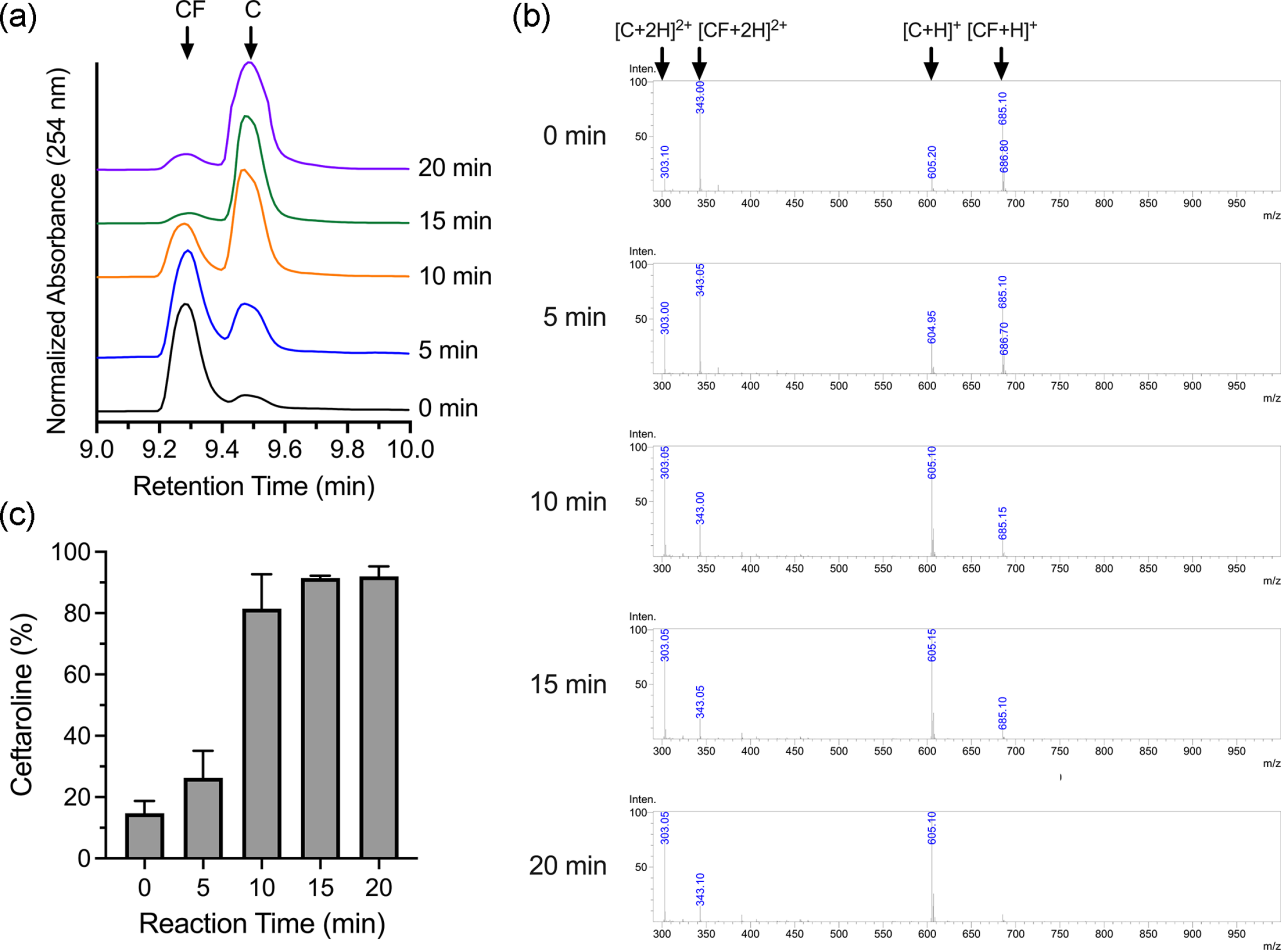
LC-MS analysis of ceftaroline fosamil thermal degradation to the ceftaroline free base. (a) Representative liquid chromatograms of the thermal hydrolysis time course of ceftaroline fosamil (CF) conversion to ceftaroline free base (C) for the retention time of 9–10 min. (b) Representative ESI-MS spectra corresponding to the liquid chromatogram retention time of 9.2–9.6 min for the thermal hydrolysis time course. Peaks are labelled with the charge state they represent for ceftaroline fosamil (CF) and ceftaroline free base (C). (c) Quantification of ceftaroline production in the thermal hydrolysis time course.

We hypothesized that exposure to high temperature for short durations might improve conversion rates while avoiding unwanted side reaction products. A timed exposure analysis confirms that exposure to 90 °C for 15 min results in ~50% conversion of Teflaro^®^ to the ceftaroline free base. Further incubation did not increase recovery (Table 2). Furthermore, LC-MS data demonstrate that the material is at least 90% pure ceftaroline free base (Fig. 2).

**Table 2.** Rapid thermal degradation of Teflaro^®^. Clinical powder equivalent to 20 mg of ceftaroline free base was resuspended in sterile water for injection and incubated at the indicated temperature for the indicated duration. Images are of lyophilized, water-insoluble precipitates

Duration (min)	Temp. (°C)	Dried product (mg)	Per cent recovery	Description
5	90	0.7, 0.6, 0.5	3±0.5%	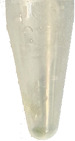
10	90	3.0, 2.8, 2.8	14.3±0.6%	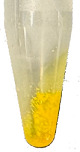
15	90	10.6, 10.3, 12.4	55.5±5.7%	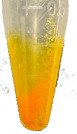
20	90	11.2, 10.3, 10.8	53.8±2.3%	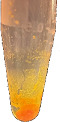

### Validation of ceftaroline by analytical LC-MS

**Fig. 3.**
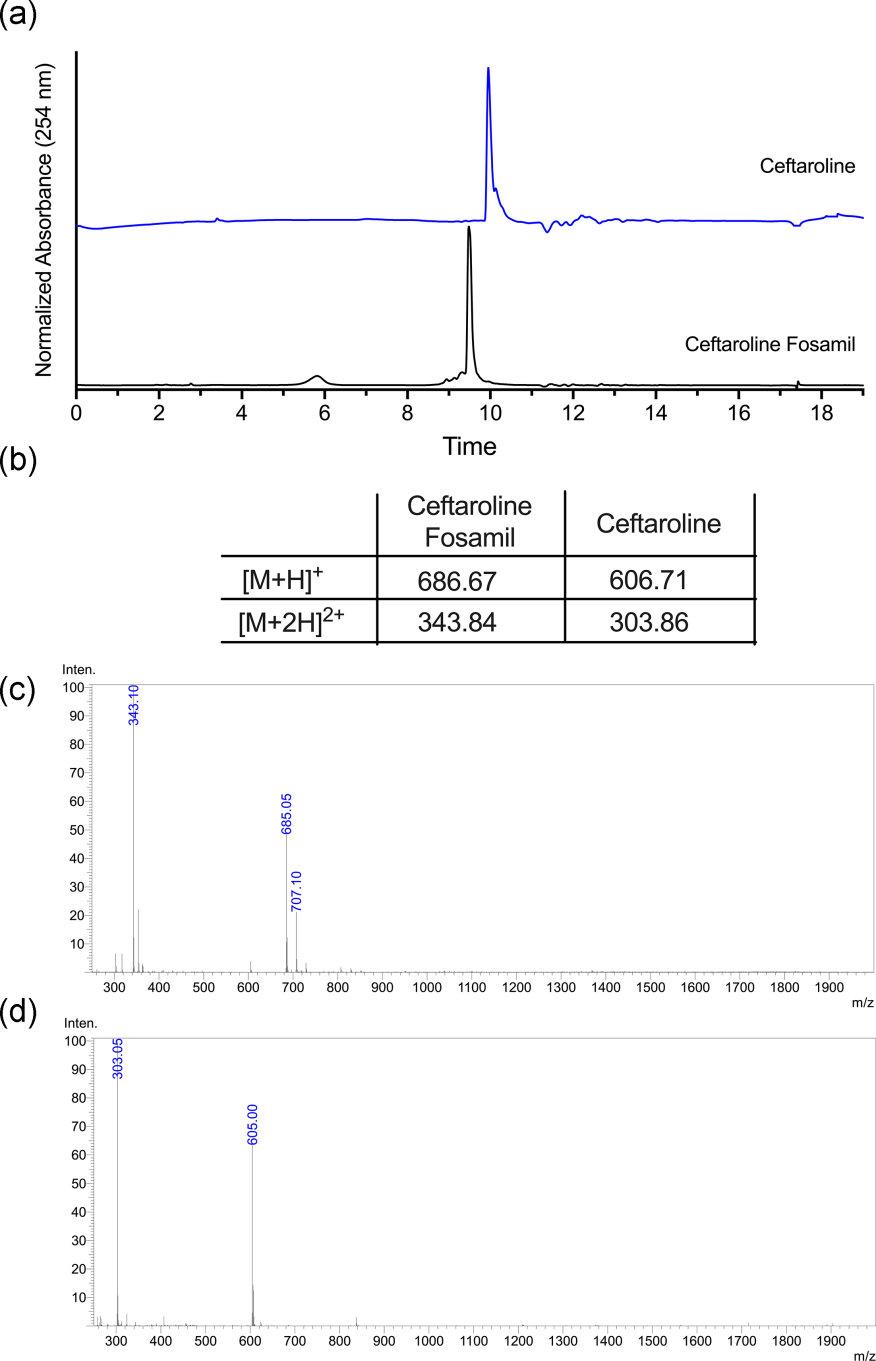
LC-MS analysis of ceftaroline fosamil thermal degradation to the ceftaroline free base. (**a**) Representative liquid chromatograms of ceftaroline fosamil (CF) and ceftaroline free base (C) standards. (**b**) Mass ions [*m/z* (amu)] observed in the ESI-MS spectra for ceftaroline fosamil and ceftaroline. (**c**) Representative ESI-MS spectra corresponding to the liquid chromatogram retention time of 9.4–9.6 min for the ceftaroline fosamil. (**d**) Representative ESI-MS spectra corresponding to the liquid chromatogram retention time of 9.9–10.1 min for the ceftaroline.

The conversion of ceftaroline fosamil (prodrug) to ceftaroline (active form and degradation product) was monitored using LC-MS to determine the composition of the thermal hydrolysis reaction. Our methodology was first confirmed by analysing the standards of ceftaroline fosamil and ceftaroline. Each standard displayed a single peak with a distinct retention time in the liquid chromatograms (Fig. 3a). Ceftaroline fosamil displayed a single peak at a retention time of 9.5 min, which shifted to 9.96 min for ceftaroline due to the loss of the phosphate group (Fig. 3a). The identity of individual compounds in the LC peaks was confirmed by MS, with ceftaroline fosamil having detectable +1 (*m/z*=685) and +2 charge states (*m/z*=343), while ceftaroline displayed the +1 (*m/z*=605) and +2 charge states (*m/z*=303), consistent with the loss of phosphate (Fig. 3b–d).

Following standardization of our method, the conversion of ceftaroline fosamil (prodrug) to ceftaroline (active form and degradation product) was monitored from the reaction mixture at various time points. Liquid chromatograms at each time point displayed two peaks detected between retention times of 9 and 10 min, demonstrating that the reaction mixture contained only two species (Figs 2a and S2). Similar to the standards, ceftaroline fosamil displayed a retention time of 9.28 min, and the identity was confirmed by ESI-MS with the same detectable ion pattern as the standard (Fig. 2a, b). Ceftaroline also displayed similar retention at 9.49 min and detectable ions as its standard (Fig. 2a, b). Analysis of the reaction time course showed that ceftaroline was present in the ceftaroline fosamil stock at 14.7% of the material. Previous reports have shown the instability of ceftaroline fosamil during storage, where the loss of the phosphate group is detectable [22]. With heat exposure, the loss of the phosphate group from ceftaroline fosamil increased significantly within 10 min and saturated to >95% by 15 min (Fig. 2c). This coincided with a shift in the retention time and the disappearance of the ceftaroline fosamil +1 charged ion in the mass spectra. Additionally, no other degradation products were observed in the liquid chromatograms or mass spectra, demonstrating the purity of the isolated reaction samples (Fig. 2a, b).

### Functional validation of ceftaroline by bioassay

In order to demonstrate that the purified ceftaroline free base retained biological activity, we performed a broth microdilution bioassay comparing the purified product to extemporaneously prepared Teflaro^®^ clinical powder. This form of bioassay using a surrogate antimicrobial compound is frequently the recommended way for a clinical lab to determine susceptibility to a novel antibiotic when a CLIA-certified susceptibility test is not available [8,9]. It is also considered the gold standard for antimicrobial susceptibility testing by the Clinical Lab Standards Institute [18]. The results of broth microdilution bioassay testing are summarized in Table 3. As anticipated, the phosphoramidated prodrug is ~32-fold less potent than purified ceftaroline. Measured ceftaroline MICs of 0.5 µg ml^−1^ (range: 0.5–0.5 µg ml^−1^) and 8 µg ml^−1^ (range: 8–8 µg ml^−1^) are consistent with published values of 0.25–0.5 µg ml^−1^ and 4–8 µg ml^−1^ for ATCC^®^ 29213 and ATCC^®^ BAA-2686, respectively [18,19].

**Table 3.** MIC of Teflaro^®^ (TEF) and purified ceftaroline (CPT) against staphylococci. Experiments were performed as analytical duplicates of three distinct inoculum preparations originating from isolated colonies per CLSI guidelines

Strain	TEF*	CPT
ATCC^®^ 29213	16	0.5
ATCC^®^ BAA-2686	≥64	8

*Microgram per millilitre.

### Conclusions, limitations and future directions

The identification of new indications and dosing guidelines for Teflaro^®^ (ceftaroline fosamil) and other phosphoramidate prodrugs is currently limited due to the inaccessibility of the active compounds. This protocol outlines a method for converting Teflaro^®^ (ceftaroline fosamil) into ceftaroline free base through a forced-degradation, non-enzymatic pathway, involving exposure of ceftaroline fosamil to a temperature of 90 °C for 15 min, which results in nearly complete conversion. The resultant product (ceftaroline free base) was analysed via LC-MS, confirming that it is at least 90% pure ceftaroline free base and retains its biological activity, demonstrating anticipated MICs against ATCC^®^ 29213 and ATCC^®^ BAA-2686 when compared to Teflaro^®^ clinical powder. Unfortunately, this method of forced degradation to active metabolites has limitations and cannot be universally applied to all cases of phosphoramidate prodrugs. Antiretrovirals with complex chemistries such as tenofovir disoproxil fumarate (Viread^®^) and anticoronovirals such as remdesivir (Veklury^®^) have multiple thermal degradation products and would require additional enzymatic transformations after dephosphoramidation to generate the biologically active agent [23,24]. However, for thermostable phosphoramidate prodrugs with well-characterized decomposition products, this method represents an attractive alternative to commercial phosphatase enzymes to access and purify the active metabolite for *in vitro* testing.

## Supplementary material

10.1099/jmm.0.002077 Uncited Supplementary Material 1.

## Abbreviations

ATCC: American Type Culture Collection
CLIA: Clinical Laboratory Improvement Amendments
CLSI: Clinical and Laboratory Standards Institute
ESI-MS: electrospray ionization-mass spectrometry
FDA: United States Food and Drug Administration
