# Supplementary material for: Rapid deprotection and purification of ceftaroline free base from its phosphoramidate prodrug
Source: J Med Microbiol. 2025 Sep 17;74(9):002077. doi: 10.1099/jmm.0.002077 (PMC12444786; doi:10.1099/jmm.0.002077)
Supplement: Uncited Supplementary Material 1. [file jmm-74-02077-s001.pdf]

Supplementary Figures

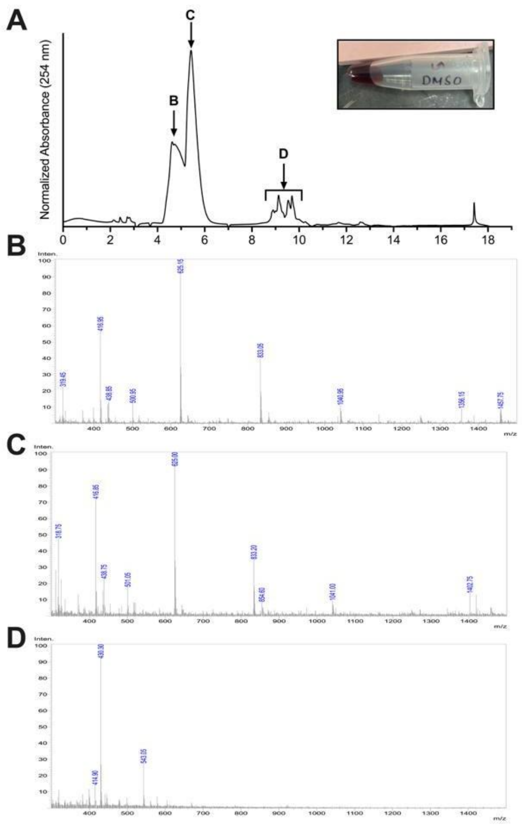

Figure S1. LC-MS analysis of the red product produced from thermal degradation of ceftaroline fosamil at 60°C for 3 hours. A) Representative full liquid chromatogram of the red product from thermal hydrolysis of ceftaroline fosamil (insert). **B)** Representative ESI-MS spectra corresponding to the liquid chromatogram retention time 4.23-5.16 min (B). **C)** Representative ESI-MS spectra corresponding to the liquid chromatogram retention time 5.16-6.03 min (C). **D)** Representative ESI-MS spectra corresponding to the liquid chromatogram retention time 8.69-10.06 min (D).

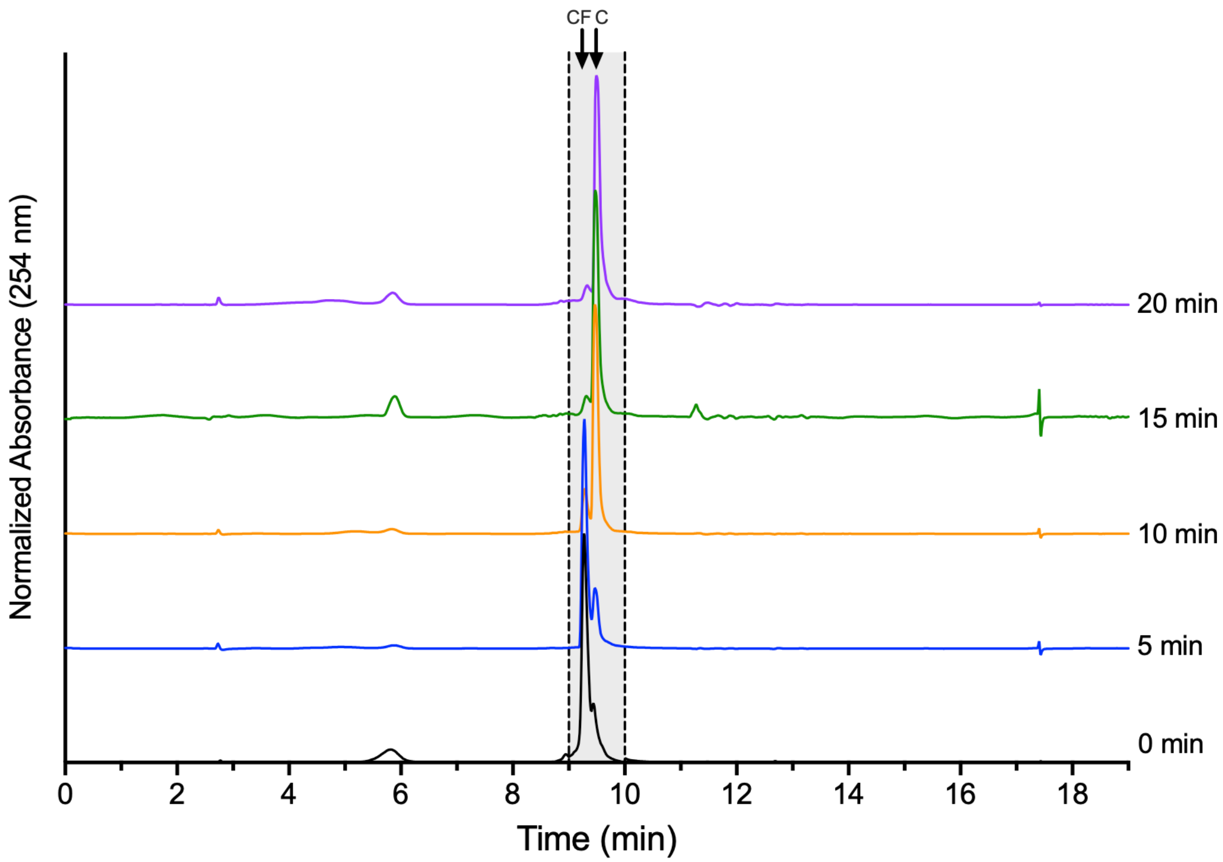

Figure S2. Full LC-MS analysis of ceftaroline fosamil thermal degradation to the ceftaroline free base. Representative full liquid chromatograms of the thermal hydrolysis time course of ceftaroline fosamil (CF) conversion to ceftaroline free base (C). Gray box highlights the region zoomed in, corresponding to **Figure 3A**.
